# Microscopy detection and molecular characterisation of *Giardia duodenalis* infection in outpatients seeking medical care in Egypt

**DOI:** 10.3389/fpubh.2024.1377123

**Published:** 2024-04-05

**Authors:** Ehab Kotb Elmahallawy, Ahmed Gareh, Marwa M. I. Ghallab, Pamela C. Köster, Alejandro Dashti, Dina Aboelsoued, Nagwa Ibrahim Toaleb, Hind Alzaylaee, Moisés Gonzálvez, Amira A. Saleh, Alaa S. Alhegaili, Ahmed Fathy Eldehn, Carolina Hernández-Castro, Begoña Bailo, David González-Barrio, David Carmena

**Affiliations:** ^1^Department of Zoonoses, Faculty of Veterinary Medicine, Sohag University, Sohag, Egypt; ^2^Departamento de Sanidad Animal, Grupo de Investigación en Sanidad Animal y Zoonosis (GISAZ), Universidad de Córdoba, Córdoba, Spain; ^3^Department of Parasitology, Faculty of Veterinary Medicine, Aswan University, Aswan, Egypt; ^4^Department of Medical Parasitology, Faculty of Medicine, Kafrelsheikh University, Kafr El Sheikh, Egypt; ^5^Parasitology Reference and Research Laboratory, National Centre for Microbiology, Majadahonda, Spain; ^6^Faculty of Health Sciences, Alfonso X El Sabio University (UAX), Villanueva de la Cañada, Madrid, Spain; ^7^Faculty of Medicine, Alfonso X El Sabio University (UAX), Villanueva de la Cañada, Madrid, Spain; ^8^Department of Parasitology and Animal Diseases, Veterinary Research Institute, National Research Centre, Cairo, Egypt; ^9^Department of Biology, College of Science, Princess Nourah bint Abdulrahman University, Riyadh, Saudi Arabia; ^10^Departamento de Sanidad Animal, Facultad de Veterinaria, Campus de Excelencia Internacional Regional “Campus Mare Nostrum”, Universidad de Murcia, Murcia, Spain; ^11^Department of Medical Parasitology, Faculty of Medicine, Zagazig University, Zagazig, Egypt; ^12^Department of Medical Laboratory, College of Applied Medical Sciences, Prince Sattam bin Abdul Aziz University, Alkharj, Saudi Arabia; ^13^Department of Otorhinolaryngology, Kasr Al-Ainy Medical School, Cairo University, Cairo, Egypt; ^14^Parasitology Group, Faculty of Medicine, Academic Corporation for the Study of Tropical Pathologies, University of Antioquia, Medellín, Colombia; ^15^Centre for Biomedical Research in Infectious Diseases (CIBER), Carlos III Health Institute (ISCIII), Madrid, Spain

**Keywords:** *Giardia*, conventional microscopy, epidemiology, diarrhoea, human, assemblage, PCR

## Abstract

**Introduction:**

Giardiosis remains one of the most prevalent enteric parasitic infections globally. Earlier molecular-based studies conducted in Egypt have primarily focused on paediatric clinical populations and most were based on single genotyping markers. As a result, there is limited information on the frequency and genetic diversity of *G. duodenalis* infections in individuals of all age groups.

**Methods:**

Individual stool samples (*n* = 460) from outpatients seeking medical care were collected during January–December 2021 in Kafr El-Sheikh governorate, northern Egypt. Initial screening for the presence of *G. duodenalis* was conducted by coprological examination. Microscopy-positive samples were further confirmed by real-time PCR. A multilocus sequence typing approach targeted amplification of the glutamate dehydrogenase (*gdh*), beta-*giardin* (*bg*), and triose phosphate isomerase (*tpi*) genes was used for genotyping purposes. A standardised epidemiological questionnaire was used to gather basic sociodemographic and clinical features of the recruited patients.

**Results:**

*Giardia duodenalis* cysts were observed in 5.4% (25/460, 95% CI: 3.6–7.9) of the stool samples examined by conventional microscopy. The infection was more frequent in children under the age of 10 years and in individuals presenting with diarrhoea but without reaching statistical significance. Stool samples collected during the winter period were more likely to harbour *G. duodenalis*. All 25 microscopy-positive samples were confirmed by real-time PCR, but genotyping data was only available for 56.0% (14/25) of the isolates. Sequence analyses revealed the presence of assemblages A (78.6%, 11/14) and B (21.4%, 3/14). All assemblage A isolates were identified as sub-assemblage AII, whereas the three assemblage B sequences belonged to the sub-assemblage BIII. Patients with giardiosis presenting with diarrhoea were more frequently infected by the assemblage A of the parasite.

**Conclusion:**

This is one of the largest epidemiological studies evaluating *G. duodenalis* infection in individuals of all age groups in Egypt. Our molecular data suggest that *G. duodenalis* infections in the surveyed population are primarily of anthropic origin. However, because assemblages A and B are zoonotic, some of the infections identified can have an animal origin. Additional investigations targeting animal (domestic and free-living) and environmental (water) samples are warranted to better understand the epidemiology of giardiosis in Egypt.

## 1 Introduction

Giardiosis by *Giardia duodenalis* (syn. *G. lamblia, G. intestinalis*) is the most frequently reported intestinal protozoan infection worldwide, with an estimated 280 million people being infected each year (1). The pathogen has a ubiquitous distribution, although most human cases occur predominantly in humid and temperate regions (2–4). As other members of the *Giardia* genus, *G. duodenalis* has a direct life cycle (monoxenus) without intermediate hosts. Infection occurs by ingestion of the transmissive cyst stage, either directly through contact with infected individuals or animals or indirectly from food or water contaminated with faecal material. In the duodenum, excystation releases vegetative trophozoites (two per cyst) into the small intestinal lumen, where they divide by binary fission and attach to the host epithelium via a ventral adhesive disc. Encystation of the trophozoite to the cyst stage occur in the colon and is promoted by exposure to bile. The life cycle is completed when cysts are excreted into the environment with the faeces of infected hosts (5). *Giardia duodenalis* cysts are environmental-resistant and can stay viable for prolonged periods of time under appropriate conditions of humidity and temperature. *Giardia duodenalis* cysts are also resistant to chemical disinfectants (e.g., chlorine and ozone) commonly used in the water industry (6, 7).

The clinical manifestations of human giardiosis vary from asymptomatic carriage to a wide diversity of symptoms including acute or chronic diarrhoea, dryness, abdominal discomfort, nausea, vomiting, flatulence, weight loss, and anaemia resulting from vitamin A, iron, and zinc deficiencies (8–10). In poor-resource areas giardiosis has been associated with impairment of growth and cognitive development during childhood (11). The pathogenic effect of giardiosis occurs through multiple mechanisms including functional disruption of the epithelial barrier, shortening of brush border microvilli, gut microbiota dysbiosis, apoptosis of enterocytes, electrolyte/nutrient/water malabsorption, and anion hypersecretion, among others (12, 13). The severity of the disease is possibly dogged by multiple factors such as the virulence of the parasite (14), the immunological and nutritional status of the host (15), the occurrence of coinfection with other pathogens (16), and the frequency of cases refractory to treatment (17). Young children, the older adults, and immunocompromised individuals are more susceptible to the infection (18, 19). In developing countries, infections by *G. duodenalis* are concomitant with poor sanitary conditions, poor water quality, and overcrowding (20, 21).

Diagnosis of giardiosis is primarily conducted by microscopy analysis of stool samples in most clinical microbiology laboratories, particularly in poor-resource settings (22). In recent years, PCR-based methods have been increasingly shown to be sensitive and cost-effective alternatives to microscopy examination of stools. Advantages of molecular assays over conventional microscopy include highly sensitive and specific identification of parasite DNA and rapid turnover with little hands-on work (23–26). When coupled to Sanger or next-generation amplicon sequencing, PCR methods allow species and genotype confirmation (27). Nucleotide sequence analyses of the small subunit ribosomal RNA (*ssu* rRNA), glutamate dehydrogenase (*gdh*), beta-giardin (*bg*), and triose phosphate isomerase (*tpi*) genes have evidenced that *G. duodenalis* is indeed a multi-species complex composed of eight (A-H) assemblages that differ widely in host range and specificity, with assemblages A and B responsible for ~95% of human infections (4, 28). Canine-adapted assemblages C-D, feline-adapted assemblage F, and ungulate-adapted assemblage E can occasionally cause human infections (4, 28). Giardiosis is endemic in Egypt. Human infections have been estimated at 9.2–80.2% by conventional microscopy, at 19.0–38.4% by ELISA, at 7.9% by rapid diagnostic tests, and at 11.5–42.3% by PCR (Table 1) (29–53). Most of the epidemiological studies were carried out on paediatric populations presenting with gastrointestinal manifestations. Molecular studies demonstrated that assemblage B (55.3%, 587/1,061) is more prevalent than assemblage A (36.9%, 392/1,061), with assemblage E (2.7%, 29/1,061), assemblage C (0.1%, 1/1,061) and mixed (4.9%, 52/1,061) infections being also detected at much lower rates (Table 1) (29–53). This study aimed to determine the prevalence and molecular diversity of *G. duodenalis* in outpatients seeking medical care in northern Egypt. To do so, conventional microscopy was used as screening method and molecular (PCR and Sanger sequencing) methods as confirmatory and genotyping methods.

**Table 1:** Occurrence and genetic diversity of *Giardia duodenalis* in human populations in Egypt.

Location	Governorate	Age group	Clinical symptoms	Detection method	Infection rate (%)	Pos./Total	Assemblage (*n*)	Sub- Assemblage (*n*)	Reference
Lower Egypt	Cairo	All	Yes	CM	15.5	62/400	–	–	(29)
				PCR	15.0	60/400	A (22), B (38)	ND	
		All	Yes	CM	30.9	30/97	–	–	(30)
				PCR	42.3	41/97	A (31), B (8), A + B (2)	AI (**24**), AII (**7**)	
		Paediatric	Yes	PCR^a^	–	92/96	A (21), B (54)	AII (21)	(31)
		Paediatric	Yes	CM	8.0	8/100	–	–	(31)
				ELISA	19.0	19/100	–	–	
				PCR	26.0	26/100	ND	ND	
		Paediatric	Yes	CM	18.8	33/176	–	–	(32)
				PCR	13.6	24/176	A (4), B (20)	ND	
		Paediatric	Yes	CM	15.5	184/1,187	–	–	(33)
				PCR	18.9	224/1,187	A (42), B (182)	ND	
	Dakhalia	All	Both	CM	17.8	33/185	–	–	(34)
				ELISA	38.4	71/185	–	–	
	El-Beheira	Paediatric	Yes	CM	24.0	24/100	–	–	(35)
				PCR	–	16/24	A (16)	AII (16)	
	Ismaillia	Paediatric	Yes	CM	9.2	12/130	–	–	(36)
				PCR	–	15/15	A (1), B (12), A + B (1), C (1)	AI/AII (1), BIII (1), BIV (1), BIII/BIV (2)	
		Paediatric	Yes	RDT	7.9	13/165	–	–	(37)
				PCR	21.2	35/165	A (5), B (14), E (1), A/B (1), A/E (1)	AII (5)	
		Paediatric	Both	CM	9.9	65/660	–	–	(38)
				PCR	–	60/65	A (40), B (18), A + B (2)	AI (36), AII (4)	
	Kafr El Sheikh	All	Both	CM	56.9	181/318	–	–	(39)
				PCR	–	65/65	A (26), B (32), A + B (7)	AII (2), BIII (19), BIV (1)	
		NS	NS	PCR^a^	–	48	A (16), B (32)	AI (2), AII (3)	(41)
	Several	All	NS	PCR^a^	–	18/52	A (1), B (14), E (3)	ND	(40)
		Paediatric	Both	CM	29.2	47/161	–	–	(41)
				PCR^a^	–	35/47	A (27), B (8)	AII (27), BIII (8)	
		Paediatric	Both	PCR	11.3	66/585	A (31), B (34), A + B (1)	AII (31)	(44)
	Sharkia	Paediatric	Both	ELISA	41.3	62/150	–	–	(42)
		Paediatric	Both	CM	9.8	61/617	–	–	(43)
				PCR	–	37/61	A (7), B (30)	ND	
		All	Both	CM	17.9	17/95	–	–	(44)
	Suez Canal	Paediatric	Both	CM	13.5	88/650	–	–	(45)
				PCR	–	88/88	A (50), B (36), A + B (2)	AI (29), AII (20), AI + AII (1)	
	West Delta	Paediatric	Both	CM	18.1	57/315	–	–	(46)
				PCR	–	57/57	A (9), B (21), A + B (27)	ND	
Upper Egypt	Assiut	Paediatric	Yes	CM	38.6	27/70	–	–	(47)
				PCR	–	22/27	B (22)	ND	
		Paediatric	Both	CM	24.2	40/165	–	–	(48)
				PCR	–	35/40	A (16), B (11), A + B (8)	–	
	Beni-Suef	Paediatric	Yes	CM	27.7	36/130	–	–	(49)
				PCR	–	28/36	A (3), B (25)	AII (3), BIII (4), BIV (5)	
	Fayium	Paediatric	Both	PCR^a^	–	25/40	E (25)	–	(53)

CM, Conventional microscopy; ELISA, Enzyme linked immunosorbent assay; ND, Not determined; NS, Not specified; PCR, Polymerase chain reaction; RDT, Rapid diagnostic test.

a Molecular study based on selected *Giardia*-positive samples.

## 2 Materials and methods

### 2.1 Ethical statement

The study was conducted in accordance with the Declaration of Helsinki, and approved by the Kafrelsheikh University Research Ethics Committee (protocol code MKSU 50–1-10). Signed informed consents were obtained from all patients that volunteer to participate in the study. Stool samples and associated epidemiological and clinical data were anonymized to protect the privacy of the participants.

### 2.2 Patient recruitment and sample collection

Single fresh stool specimens from outpatients seeking medical attention at the Kafrelsheikh University Hospital (Kafr El-Sheikh governorate, Egypt) were collected during the period January–December 2021 without specific exclusion criteria. Each stool sample was collected in a screw-capped container and labelled with a unique identifier code. Information regarding sex, age, location, and sampling date were also recorded. Faecal consistency was categorised as either diarrheic or formed. Samples were submitted for microscopy examination within 3 h of collection.

### 2.3 Microscopy examination

#### 2.3.1 Direct wet smear

Direct saline (0.85% NaCl) and Lugol’s iodine wet mounts were prepared from freshly passed stool specimens for the detection of *G. duodenalis* cysts and trophozoites according to standard procedures (54). Cover slips were gently put over microscopy glass slides to spread out the emulsion and then examined under a light microscope using a low-power (10×) objective for screening and a high-power (40×) objective for confirmation of presumptive and positive findings.

#### 2.3.2 Concentration using the formalin-ethyl acetate sedimentation method

The formyl-ether concentration method was used according to recommended procedures (54). Briefly, 4 g of fresh faecal material was homogenised in 10 mL of 10% formalin and sieved through surgical gauze as a mechanical filter to remove faecal debris. The sieved suspension was transferred into a clean 15 mL centrifuge tube and 3 mL of ethyl acetate were added. After vigorous shaking, the mixture was centrifuged for 10 min at 500 × g. The supernatant was carefully discarded, and faecal smears made from the sediment and examined as described in sub-section 2.3.1.

#### 2.3.3 Concentration using the zinc sulphate flotation method

The zinc sulphate flotation method was used according to recommended procedures (54). Briefly, 4 g of fresh faecal material was homogenised in 10 mL of 10% formalin and sieved through surgical gauze as a mechanical filter to remove faecal debris. The sieved suspension was transferred into a clean 15 mL centrifuge tube and another 10 mL of 10% formalin were added. The mix was centrifuged for 10 min at 500 × g. After discarding the supernatant, a 33% zinc sulphate solution (specific gravity: 1.18) was added to the sediment, followed by homogenisation and centrifugation for 2 min at 500 × g. Faecal smears were made with 1–2 drops of the surface film and examined as described in sub-section 2.3.1.

Aliquots of all *Giardia*-positive stool samples at microscopy examination were stored in 70% ethanol and shipped to the National Centre for Microbiology, Health Institute Carlos III (Majadahonda, Spain) for downstream molecular testing.

### 2.4 DNA extraction and purification

Genomic DNA was isolated from about 200 mg of faecal samples yielding positive results for *G. duodenalis* at microscopy examination by using the QIAamp DNA Stool Mini Kit (Qiagen, Hilden, Germany) according to the manufacturer’s instructions. Extracted and purified DNA samples were eluted in 200 μL of PCR-grade water and kept at 4°C until subsequent molecular testing.

### 2.5 Molecular identification and characterization of *Giardia duodenalis*

Detection of *G. duodenalis* DNA was achieved using a real-time PCR (qPCR) method targeting a 62-bp region of the gene codifying the small subunit ribosomal RNA (*ssu* rRNA) gene of the parasite (55). For assessing its molecular diversity at the assemblage and sub-assemblage levels, we adopted a sequence-based multilocus genotyping (MLST) scheme targeting the genes encoding for the glutamate dehydrogenase (*gdh*), β-giardin (*bg*), and triose phosphate isomerase (*tpi*) proteins of *G. duodenalis*. To maximise the options of amplification success at these markers, only *Giardia* isolates that tested positive by qPCR and yielded cycle threshold (C_T_) values <32 were tested for genotyping purposes. A semi-nested PCR was used to amplify a 432-bp fragment of the *gdh* gene (56), and nested PCRs were used to amplify 511 and 530 bp fragments of the *bg* and *tpi* genes, respectively (57, 58).

### 2.6 PCR and gel electrophoresis standard procedures

Information regarding the oligonucleotides and PCR cycling conditions used for the detection and genotyping of *G. duodenalis* is summarised in Supplementary Tables 1, 2. The qPCR protocol described above was carried out on a Corbett Rotor Gene^™^ 6,000 real-time PCR system (Qiagen), with reaction mixes containing 2× TaqMan^®^ Gene Expression Master Mix (Applied Biosystems, CA, United States). All the semi-nested and nested PCR procedures mentioned above were performed on a 2720 Thermal Cycler (Applied Biosystems). Reaction mixes consistently included 2.5 units of MyTAQ^™^ DNA polymerase (Bioline GmbH, Luckenwalde, Germany) and 5–10 μL of MyTAQ^™^ Reaction Buffer containing 5 mM dNTPs and 15 mM MgCl_2_. Laboratory-confirmed positive and negative DNA samples of human origin were routinely used as controls and included in each round of PCR. PCR amplicons were visualised on 1.5% D5 agarose gels (Condalab, Madrid, Spain) stained with Pronasafe (Condalab) nucleic acid staining solutions.

### 2.7 Sequence analyses

Amplicons of the anticipated size were directly sequenced in both directions using the corresponding internal primer sets (Supplementary Table 1) in 10 μL reactions. DNA sequencing was conducted by capillary electrophoresis using the BigDye^®^ Terminator chemistry (Applied Biosystems) on an ABI 3730xl sequencer analyser (Applied Biosystems). Raw sequencing data was examined with Chromas Lite version 2.1 software^1^ to generate consensus sequences. These sequences were compared with reference sequences deposited at the National Centre for Biotechnology Information (NCBI) using the BLAST tool.^2^ Representative nucleotide sequences generated in this study have been deposited in GenBank under accession numbers PP035393–PP035398 (*gdh* locus), PP035399–PP035400 (*bg* locus), and PP035401 (*tpi* locus).

### 2.8 Phylogenetic analyses

To analyse the phylogenetic relationship among *G. duodenalis* assemblages and sub-assemblages at the *gdh*, *bg*, and *tpi* markers, maximum-likelihood trees were constructed using MEGA version 11 (59), based on substitution rates calculated with the general time reversible model and gamma distribution with invariant sites (G + I). Bootstrapping with 1,000 replicates was used to determine support for the clades.

### 2.9 Statistical analyses

Associations between *G. duodenalis* infections and potential risk factors (sex, age, sampling season, and stool consistency) were evaluated by bivariate analysis using the Pearson’s chi-square test or Fisher’s test, as required. The significance level was set at 0.05. All statistical analyses were performed using R free software (60).

## 3 Results

In the present survey, 460 individual stool samples were collected from outpatients seeking medical care. The male/female ratio was 0.84. Participating patients had a median age of 13 years (0.1–60; standard deviation: 18.8). Near half of the participating patients were children under the age of 10 (44.8%, 206/460) and presented with diarrhoea (54.1%, 249/460; Table 2).

**Table 2:** Frequency of *Giardia duodenalis* infections and genotypes according to the gender, age group, stool consistency, and sampling season of the surveyed human population (*n* = 460).

Variable	Samples (*n*)	Positive (*n*)	Frequency (%)	*p* value	Assemblage A (*n*)	Assemblage B (*n*)
Gender						
Male	210	12	5.7	0.971	6	1
Female	250	13	5.2		6	2
Age group (yrs.)						
0–10	206	16	7.8	0.967	8	1
11–35	150	9	6.0		4	2
36–60	104	0	0.0		0	0
Stool consistency						
Formed	211	7	3.3	0.102	3	1
Diarrheic	249	18	7.2		9	2
Season						
Summer	136	5	3.7	**0.027**	3	
Autumn	69	0	0.0		0	0
Winter	137	12	8.8		6	0
Spring	118	8	6.8		3	3

Bolded values indicate statistical significance.

### 3.1 Microscopy

Overall, 5.4% [25/460, 95% Confidence Interval (CI): 3.6–7.9] of faecal samples examined by microscopy tested positive for *G. duodenalis*. Neither gender, age group nor stools consistency were positively associated with a higher likelihood of having a *G. duodenalis* infection. Despite this lack of statistical significance, the pathogen was more frequently found in paediatric patients under 10 years of age (7.8%) and in patients presenting with diarrhoea (7.2%). *Giardia* infections were similarly present in males and in females (5.7% vs. 5.2%, respectively). In contrast, outpatients attended during the autumn period were less likely (*p* = 0.027) to harbour the pathogen (Table 2).

### 3.2 Confirmation of *Giardia duodenalis* by qPCR

All 25 faecal DNA samples with a *Giardia*-positive result by conventional microscopy examination tested also positive by qPCR. Yielded C_T_ values ranged from 20.2 to 31.9 (median: 22.5; standard deviation: 4.0). Most (84.0%, 21/25) of the qPCR-positive samples yielded C_T_ values <30 (Table 3).

### 3.3 Genotyping and sub-genotyping of *Giardia duodenalis* isolates

The molecular diversity of the 25 *G. duodenalis*-confirmed isolates was investigated at the assemblage and sub-assemblage levels at three (*gdh*, *bg*, and *tpi*) genetic markers. Successful PCR amplifications and sequencing data were generated for 48.0% (12/25, *gdh*), 20.0% (5/25, *bg*), and 12.0% (3/25, *tpi*) of the samples investigated at the three loci (Table 3). Overall, 56.0% (14/25) of the *Giardia*-positive samples were successfully genotyped at one locus at least. MLST data at the three assessed loci was available for a single sample (4.0%, 1/25). Subtyping data at a single locus and two loci were available for 36.0% (9/25) and 16.0% (4/25) of samples, respectively. No genotyping data could be obtained for 44.0% (11/25) of the *Giardia*-positive samples. Assemblage A (44.0%, 11/25) was more prevalent than assemblage B (12.0%, 3/25). No A + B mixed infections were detected. No host-adapted assemblages of canine (C, D), feline (F), or livestock (E) origin were identified circulating in the surveyed clinical population (Table 3).

**Table 3:** Multilocus sequence typing results of the 25 *G. duodenalis*-positive human samples successfully genotyped at any of the three loci investigated in the present survey.

Sample ID	Age (yrs.)	Gender	Season	Stool consistency	C_T_ value in qPCR	*gdh*	*bg*	*tpi*	Assigned genotype
1	47	Female	Spring	Diarrheic	22.1	AII	AII	–	AII
3	30	Male	Spring	Diarrheic	21.7	AII	–	AII	AII
4	9	Female	Spring	Diarrheic	22.2	AII	–	–	AII
10	31	Female	Summer	Diarrheic	21.6	AII	AII	AII	AII
17	39	Male	Summer	Diarrheic	21.3	AII	–	–	AII
23	10	Male	Summer	Diarrheic	22.4	–	–	–	Unknown
27	32	Male	Winter	Diarrheic	20.2	AII	AII	–	AII
28	4	Female	Winter	Diarrheic	21.3	AII	–	–	AII
29	2	Female	Winter	Diarrheic	22.5	–	–	–	Unknown
42	8	Male	Winter	Diarrheic	22.5	–	AII	–	AII
48	4	Female	Winter	Formed	30.9	–	–	AII	AII
82	1	Female	Summer	Diarrheic	29.6	–	–	–	Unknown
103	9	Male	Summer	Formed	27.1	–	–	–	Unknown
107	1	Male	Spring	Diarrheic	29.5	–	–	–	Unknown
139	22	Female	Winter	Diarrheic	21.5	–	–	–	Unknown
140	55	Male	Winter	Formed	20.2	AII	–	–	AII
141	22	Female	Winter	Formed	20.2	AII	AII	–	AII
173	22	Female	Spring	Diarrheic	24.4	BIII	–	–	BIII
177	21	Female	Spring	Diarrheic	25.7	BIII	–	–	BIII
180	51	Male	Spring	Formed	23.9	BIII	–	–	BIII
187	1	Male	Spring	Diarrheic	26.7	–	–	–	Unknown
225	17	Female	Winter	Formed	31.0	–	–	–	Unknown
310	12	Male	Winter	Diarrheic	31.6	–	–	–	Unknown
399	6	Male	Winter	Diarrheic	28.8	–	–	–	Unknown
400	1	Female	Winter	Formed	31.9	–	–	–	Unknown

The age, gender, sampling season, and stool consistency of the infected individuals are also shown.

Table 4 shows the frequency and molecular diversity of *G. duodenalis* at the *gdh*, *bg*, and *tpi* loci. Out of the 12 *gdh* sequences, nine (75.0%) were assigned to the sub-assemblage AII. Of them, seven showed 100% identity with reference sequence L40510. The remaining two sequences differed from it by 1–2 single nucleotide polymorphisms (SNPs) in the form of ambiguous (double peak) positions. Sub-assemblage BIII was identified in three (25.0%) isolates, all of them differing among them and by 1–7 SNPs with reference sequence AF069059. Most of the SNPs detected correspond to clear mutations. No isolates belonging to sub-assemblage BIV were detected. All five *bg* sequences were identified as sub-assemblage AII. Four of them were identical to reference sequence AY072723, whereas the remaining one differed form it by a single SNP in the form of a double peak at chromatogram inspection. All three *tpi* sequences were identified as sub-assemblage AII and showed 100% identity with reference sequence U57897. No assemblage B isolates could be genotyped at the *bg* and *tpi* loci.

**Table 4:** Frequency and molecular diversity of *G. duodenalis* identified at the *gdh*, *bg*, and *tpi* loci in the human population under study.

Marker	Assemblage	Sub-assemblage	No. isolates	Reference sequence	Stretch	Single nucleotide polymorphisms	GenBank ID
*gdh*	A	AII	7	L40510	64–491	None	PP035393
			1	L40510	64–491	C273Y, A409R	PP035394
			1	L40510	67–491	G458K	PP035395
		BIII	1	AF069059	40–455	A55G, C99T, C141T, T147C, G150A, A164R, C309T	PP035396
			1	AF069059	40–455	A55G, C99T, C141T, T147C, G150A, C309T	PP035397
			1	AF069059	40–455	C309T	PP035398
*bg*	A	AII	4	AY072723	102–592	None	PP035399
			1	AY072723	102–594	T164Y, C540Y	PP035400
*tpi*	A	AII	3	U57897	294–805	None	PP035401

GenBank accession numbers are provided. *bg*, β-giardin; *gdh*, Glutamate dehydrogenase; K, G/T; R, A/G; *tpi*, Triose phosphate isomerase; Y, C/T.

Our statistical analyses also revealed that the distribution of assemblages A and B was independent of all the variables (gender, age group, stool consistency and sampling season) considered as potential risk factors in the present study (Table 2).

**Figure 1:**
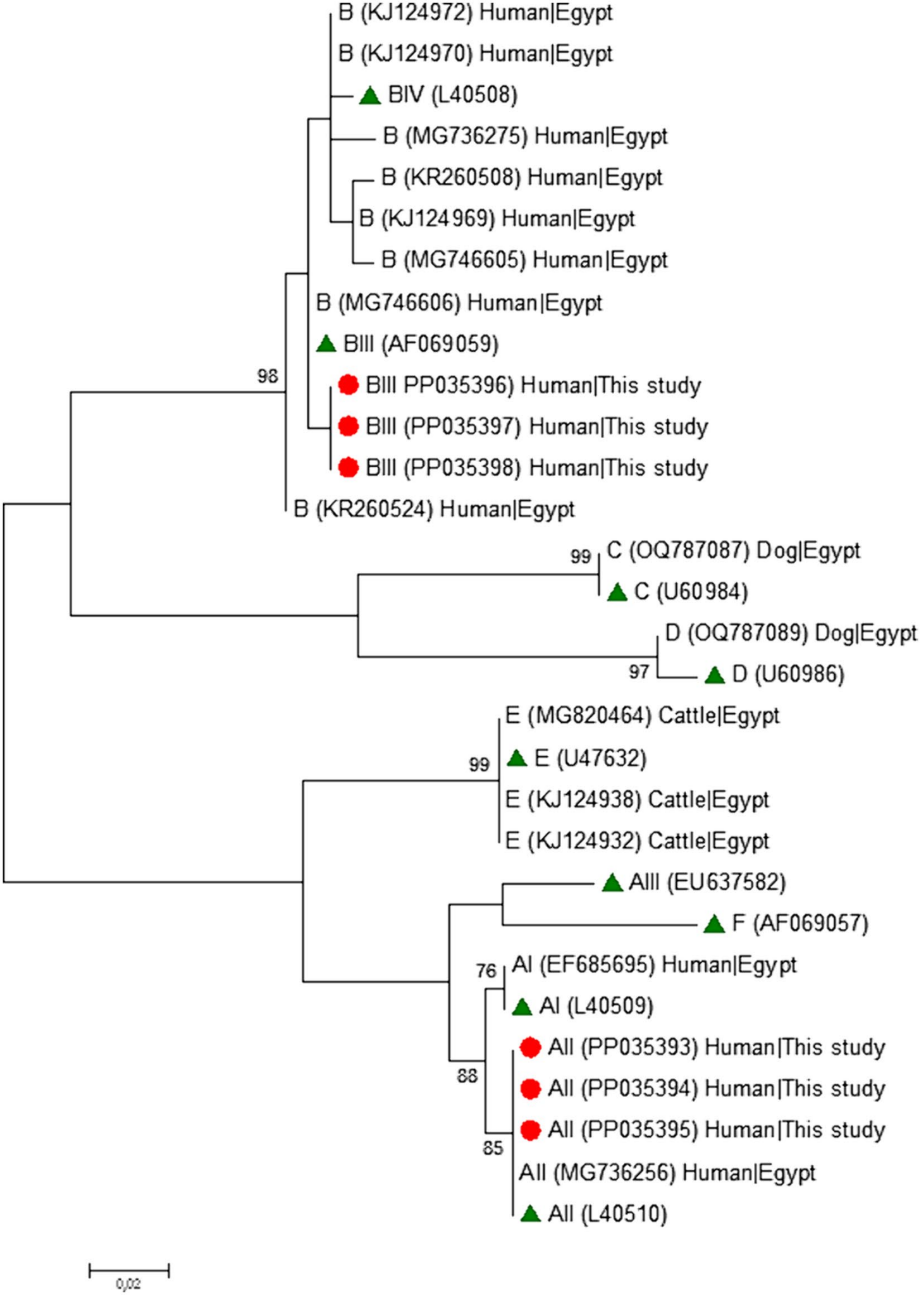
Phylogenetic relationship among *Giardia duodenalis* assemblages and sub-assemblages revealed by a maximum likelihood analysis of the partial *gdh* rDNA gene. Substitution rates were calculated by using the general time reversible model. Numbers on branches are percent bootstrapping values over 70% using 1,000 replicates. The filled red circle indicates the nucleotide sequence generated in the present study. The filled green triangles indicate reference sequences. Human and animal sequences from Egyptian origin retrieved from GenBank were included in the analysis for comparative purposes.

**Figure 2:**
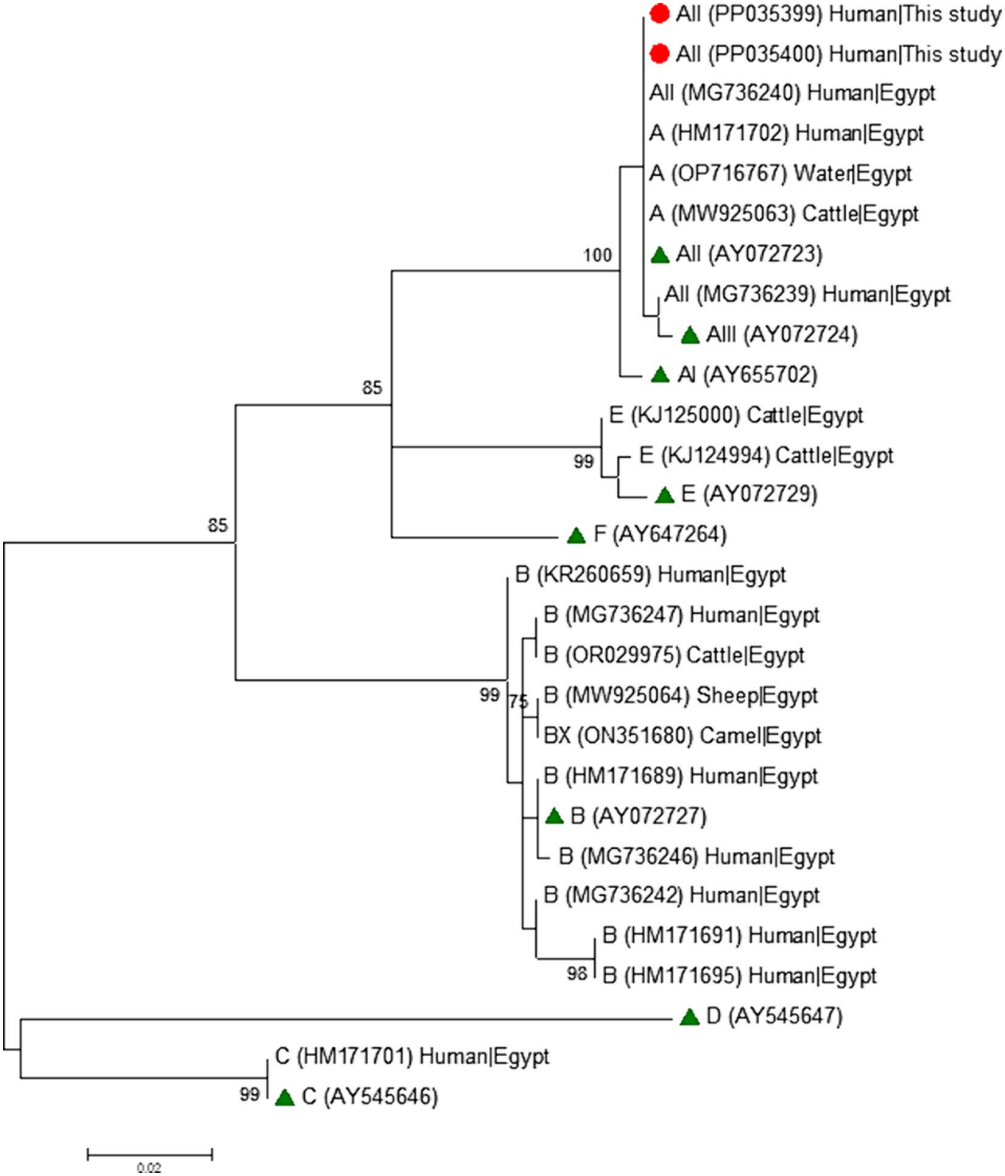
Phylogenetic relationship among *Giardia duodenalis* assemblages and sub-assemblages revealed by a maximum likelihood analysis of the partial *bg* rDNA gene. Substitution rates were calculated by using the general time reversible model. Numbers on branches are percent bootstrapping values over 70% using 1,000 replicates. The filled red circle indicates the nucleotide sequence generated in the present study. The filled green triangles indicate reference sequences. Human and animal sequences from Egyptian origin retrieved from GenBank were included in the analysis for comparative purposes.

**Figure 3:**
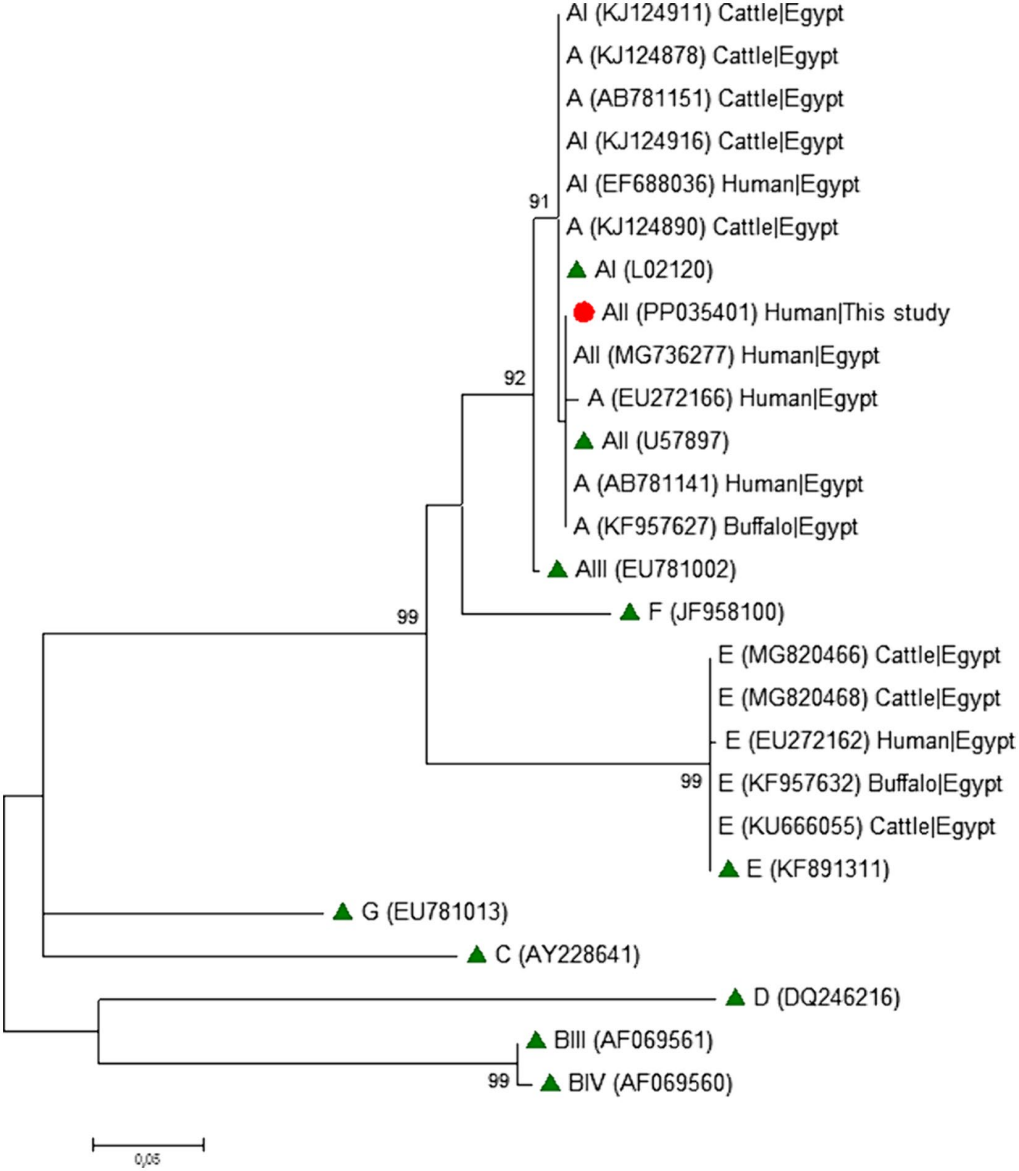
Phylogenetic relationship among *Giardia duodenalis* assemblages and sub-assemblages revealed by a maximum likelihood analysis of the partial *tpi* rDNA gene. Substitution rates were calculated by using the general time reversible model. Numbers on branches are percent bootstrapping values over 70% using 1,000 replicates. The filled red circle indicates the nucleotide sequence generated in the present study. The filled green triangles indicate reference sequences. Human and animal sequences from Egyptian origin retrieved from GenBank were included in the analysis for comparative purposes.

Phylogenetic analyses conducted at the *gdh* (Figure 1), *bg* (Figure 2) and *tpi* (Figure 3) loci clearly showed that our *G. duodenalis* sequences formed well-supported clades with appropriate reference sequences retrieved from GenBank.

## 4 Discussion

Giardiosis remains a public health concern globally, with young children living in poor-resource settings carrying the bulk of the infection. Interestingly, large case/control and longitudinal studies conducted in endemic areas (most of them targeting paediatric populations) have shown that *G. duodenalis* infection was not associated with acute diarrhoea (61) or even has a protective effect against it (62, 63). These data highlighted to the need of clarifying the circumstances under which *G. duodenalis* infection may be involved in the development of diarrheal disease (either acute or persistent) and whether specific *Giardia* genotypes exhibit enhanced pathogenicity over other genotypes (61). This study assessed the frequency and genetic diversity of *G. duodenalis* infection in outpatients seeking medical care in northern Egypt. Strengths of the survey include (i) the recruiting of patients belonging to all age groups (allowing better insight of the infection through lifespan) with and without diarrhoea (allowing the study of the infection according to clinical status and the genetic variant of the parasite) and (ii) the adoption of a MLST approach to better understand the genetic diversity of the *G. duodenalis* isolates obtained.

In the present study, *G. duodenalis* infection rates were similarly found in males and females (5.7% vs. 5.2%). This finding aligns with those previously reported in studies conducted in the Egyptian provinces of El-Dakahlia, El-Gharbia, and Damietta (52) and the West Delta region (46). Conversely, males were more likely to harbour *G. duodenalis* infections in studies conducted in Egypt (30, 48) and other Mediterranean and Middle East countries including Algeria (64), Saudi Arabia (65) and Yemen (66). Taken together, these findings suggest that behavioural and occupational factors likely play a role in the risk of human transmission and infection. Our results also indicated that *G. duodenalis* infections were more frequent in children aged less than 10 years than in other age groups. This outcome is consistent with the results obtained in other microscopy-based studies conducted in Egypt (33, 46). Young children might be more vulnerable to *G. duodenalis* infections because of their poor personal hygiene practices, higher exposure to unsanitary environments, and immature immune system compared to adults (67, 68). However, it should be noted that other surveys have reported higher parasite frequency rates in the 10–20 years age group (30).

Despite the fact that *G. duodenalis* infections were more frequently identified in individuals presenting with diarrhoea than in those without diarrhoea (7.2% vs. 3.3%), the difference was not statistically significant. Remarkably, large cohort studies conducted in sub-Saharan countries including the Global Enteric Multicenter Study (GEMS) (69), the Malnutrition and Enteric Disease Study (MAL-ED) (70), and the Vaccine Impact on Diarrhea in Africa Case–Control Study (VIDA) (71) have evidenced that *G. duodenalis* detection was more common among non-diarrheal than diarrheal infected children. The reasons for this trend are unclear, but an indirect mechanism triggered by *G. duodenalis* leading to changes in colonisation/infection by other enteric pathogens associated with moderate-to-severe-diarrhoea has been suggested (71).

Regarding environmental factors, we found that *G. duodenalis* infections were significantly more reported during the winter (January–March) months. This is in contrast with other studies conducted in Egypt where the parasite was more frequently identified during mid-summer and late winter (33). In the absence of robust temporal series of human giardiosis in Egypt, assessing the seasonal variation of the infection is a difficult task, so available data (including those from this study) should be interpreted with caution.

In the present study, all 25 *G. duodenalis*-positive samples at microscopy examination were confirmed by qPCR with C_T_ values <32, indicative of moderate-to-high parasite burdens. However, genotyping data were only available for 56.0% (14/25) of them. This moderate genotyping success rate can be explained by the fact that the *gdh*, *bg*, and *tpi* genotyping markers used in the present survey are all single-copy genes with limited diagnostic sensitivity. In contrast, the qPCR assay used here targeted the *ssu* rRNA marker, a multiple copy gene with high diagnostic sensitivity particularly suited for detection purposes (27, 28). Our genotyping analyses revealed that assemblage A was more prevalent than assemblage B (78.6% vs. 21.4%). Similar results have been reported in previous molecular surveys conducted in Cairo Governorate (30), El-Beheira Governorate (35), Ismaillia Governorate (38), and Sharkia Governorate (44) in Lower Egypt, and Assiut Governorate (48) in Upper Egypt. However, it should be noted that the opposite trend has been more frequently reported, with assemblage B accounting for 55.3% (587/1,061) of the human cases of giardiosis genotyped in the country (Table 1). No mixed A + B infections were detected in the present study, but seems a relatively common finding in other studies. Taken together, these data indicate that human *G. duodenalis* infections are primarily of anthropic nature in Egypt. However, and unknown fraction of these infections might be of animal origin, as both assemblages A and B are zoonotic. Although probably infrequent, zoonotic transmission events are also possible, as demonstrated by the occasional presence of canine-adapted assemblage C in a symptomatic children in Ismaillia Governorate (36) and hoofed-adapted assemblage E in human population from different Egyptian governorates (37, 40, 53). Unveiling the epidemiology of giardiosis in Egypt is a complex task hampered by our limited knowledge of which extent the human, animal, and environmental reservoirs contribute to the burden of human disease. Ideally, this task should be tackled under a One Health approach targeting all three reservoirs in the same spatiotemporal frame. Investigations should be directed towards the identification of sources of infection and transmission pathways including the identification of spillover events involving cross-species transmission in the wildlife-domestic interface. More research is also needed to investigate waterborne and foodborne transmission of *G. duodenalis* infections. Generating good quality molecular data is essential for these purposes.

This study unveiled variations in *G. duodenalis* assemblage frequencies among individuals of different ages and sexes. Assemblage A was more prevalently found in children less than 10 years of age. Of note, assemblage A was also the most common *G. duodenalis* genetic variant circulating in children with diarrhoea aged 2–8 years and was significantly associated with the duration and severity of the infection at Cairo Governorate (29). In contrast, children with diarrhoea below the age of 6 were more likely infected with assemblage B than with assemblage A at Beni-Suef Governorate (49). In other study conducted in Algeria, symptomatic children aged less than 7 years were predominantly infected with assemblage A, whereas assemblage B was more frequent in children older than 11 years (64). Overall, all the findings mentioned above pointed out to a mixed distribution pattern of *G. duodenalis* assemblages in Egypt regardless the geographical region of origin and the human population under study, very likely reflecting a complex epidemiological scenario characterised by multiple sources of infection and transmission pathways.

Currently there is no clear correlation between *G. duodenalis* assemblages and the outcome and severity of the infection, with limited studies on this topic (72). Large case–control studies of paediatric populations conducted in Ethiopia (73) and Mozambique (74) reported similar assemblage distributions among individuals with and without diarrhoea, suggesting that the genetic variant of the parasite is not an essential factor in the outcome of the infection. Although the present study lacks a case/control design, most children presenting with diarrhoea harboured the assemblage A of the parasite. This results is consistent with those reported in similar studies conducted in Australia (75), Bangladesh (76), Egypt (29), India (77), Iran (78), Spain (79), Syria (80), Turkey (81), and the UK (82).

This study has some design and methodological limitations that must be taken into account when evaluating the results obtained and the conclusions reached. First, the transversal nature of the study is not adequate to follow up the course of giardiosis and to capture seasonal variations of the infection. Second, initial screening of *G. duodenalis* infection was conducted by microscopy examination of a single stool sample per patient. Because of the limited sensitivity of this method, the infection rate reported here is likely an underestimation of the true one. Third, results obtained here might not be representative of other epidemiological scenarios or geographical areas in Egypt. And fourth, the relatively limited number of *G. duodenalis* isolates successfully genotyped (some of them at a single locus) might have biased the estimation of the true molecular diversity of *G. duodenalis* infections and the actual frequency of assemblages/sub-assemblages circulating in the investigated human population. This fact might have compromised the accuracy of some of the results obtained and the conclusions reached and warrants further investigations to corroborate the genotyping data presented here.

## 5 Conclusion

Endemic giardiosis continues to pose a significant public health threat in Egypt. Children under 10 years old are particularly vulnerable to the infection, so improved personal hygiene practices and promotion of healthy habits together with better access to safe drinking water and sanitary facilities are necessary to minimise the risk of transmission and infection. Our molecular findings revealed that most human cases of giardiosis were caused by assemblage A, which was the *G. duodenalis* genetic variant more prevalently found in individuals with diarrhoea. These results, together with the absence of animal-adapted assemblages C-F suggest that *G. duodenalis* infections in the surveyed population are primarily anthropic in nature. However, we cannot ruled out that an unknown proportion of the infections detected have an animal origin, as both assemblages A and B are zoonotic. Additional investigations are warranted to better understand the epidemiology of giardiosis in Egypt. Of particular interest would be conducting molecular-based epidemiological surveys in animal (domestic and free-living) and environmental (water) samples to better improve our knowledge on the sources of infection and transmission pathways of the parasite.

## Data availability statement

The datasets presented in this study can be found in online repositories. The names of the repository/repositories and accession number(s) can be found at: [GenBank under accession numbers PP035393–PP035398 (*gdh* locus), PP035399–PP035400 (*bg* locus), and PP035401 (*tpi* locus)].

## Ethics statement

The study was conducted in accordance with the Declaration of Helsinki and approved by the Kafrelsheikh University Research Ethics Committee (protocol code MKSU 50–1-10). Signed informed consents were obtained from all patients that volunteer to participate in the study. Stool samples and associated epidemiological and clinical data were anonymized to protect the privacy of the participants. The studies were conducted in accordance with the local legislation and institutional requirements. Written informed consent for participation in this study was provided by the participants’ legal guardians/next of kin.

## Author contributions

EE: Conceptualization, Data curation, Formal analysis, Funding acquisition, Investigation, Methodology, Resources, Software, Validation, Visualization, Writing – original draft, Writing – review & editing. AG: Formal analysis, Software, Supervision, Validation, Visualization, Writing – original draft, Writing – review & editing. MIG: Data curation, Formal analysis, Methodology, Software, Validation, Visualization, Writing – review & editing. PK: Formal analysis, Investigation, Methodology, Software, Validation, Visualization, Writing – original draft, Writing – review & editing. AD: Data curation, Formal analysis, Methodology, Software, Visualization, Writing – review & editing. DA: Conceptualization, Data curation, Methodology, Software, Validation, Visualization, Writing – original draft. NT: Conceptualization, Data curation, Formal analysis, Investigation, Methodology, Software, Validation, Writing – original draft. HA: Data curation, Formal analysis, Software, Validation, Visualization, Writing – original draft, Writing – review & editing. MG: Data curation, Formal analysis, Software, Validation, Visualization, Writing – original draft, Writing – review & editing. AS: Conceptualization, Data curation, Formal analysis, Resources, Software, Validation, Writing – original draft, Writing – review & editing. AA: Data curation, Formal analysis, Resources, Software, Validation, Visualization, Writing – original draft. AE: Data curation, Formal analysis, Software, Validation, Writing – original draft. CH-C: Formal analysis, Methodology, Writing – review & editing. BB: Formal analysis, Methodology, Software, Validation, Writing – review & editing. DG-B: Data curation, Formal analysis, Software, Validation, Writing – review & editing. DC: Conceptualization, Data curation, Formal analysis, Funding acquisition, Investigation, Software, Supervision, Validation, Writing – original draft, Writing – review & editing.

## Footnotes

^1^ http://chromaslite.software.informer.com/2.1

^2^ http://blast.ncbi.nlm.nih.gov/Blast.cgi
