# Supplementary material for: Microscopy detection and molecular characterisation of Giardia duodenalis infection in outpatients seeking medical care in Egypt
Source: Front Public Health. 2024 Apr 5;12:1377123. doi: 10.3389/fpubh.2024.1377123 (PMC11026549; doi:10.3389/fpubh.2024.1377123)
Supplement: Supplementary file 1 [file Table_1.DOCX]

**Supplementary Table 1.** Oligonucleotides used for the molecular identification and/or characterization of the *Giardia duodenalis* investigated in this study.

| **Locus** | **Oligonucleotide** | **Sequence (5´–3´)** | **Reference** |
| --- | --- | --- | --- |
| *ssu* rRNA | Probe | FAM–CCCGCGGCGGTCCCTGCTAG–BHQ1 | 55 |
|  | Gd-80F | GACGGCTCAGGACAACGGTT |  |
|  | Gd-127R | TTGCCAGCGGTGTCCG |  |
| *gdh* | GDHeF | TCAACGTYAAYCGYGGYTTCCGT | 56 |
|  | GDHiF | CAGTACACCTCYGCTCTCGG |  |
|  | GDHiR | GTTRTCCTTGCACATCTCC |  |
| *bg* | G7_F | AAGCCCGACGACCTCACCCGCAGTGC | 57 |
|  | G759_R | GAGGCCGCCCTGGATCTTCGAGACGAC |  |
|  | G99_F | GAACGAACGAGATCGAGGTCCG |  |
|  | G609_R | CTCGACGAGCTTCGTGTT |  |
| *tpi* | AL3543 | AAATIATGCCTGCTCGTCG | 58 |
|  | AL3546 | CAAACCTTITCCGCAAACC |  |
|  | AL3544 | CCCTTCATCGGIGGTAACTT |  |
|  | AL3545 | GTGGCCACCACICCCGTGCC |  |
|  | RD5 | ATCTGGTTGATCCTGCCAGT |  |

*bg*: β-giardin; *gdh*: Glutamate dehydrogenase; *ssu* rRNA: Small subunit ribosomal RNA; *tpi*: Triose phosphate isomerase.
