# Supplementary material for: Microscopy detection and molecular characterisation of Giardia duodenalis infection in outpatients seeking medical care in Egypt
Source: Front Public Health. 2024 Apr 5;12:1377123. doi: 10.3389/fpubh.2024.1377123 (PMC11026549; doi:10.3389/fpubh.2024.1377123)
Supplement: Supplementary file 2 [file Table_2.DOCX]

**Supplementary Table 2.** PCR cycling conditions used for the molecular identification and/or characterization of the *Giardia duodenalis* species investigated in this study.

|  |  | **Temperature and time** | | | | |  |  |  |
| --- | --- | --- | --- | --- | --- | --- | --- | --- | --- |
| **Target organism** | **Locus** | | **Initial denaturation** | **Denaturation** | **Annealing** | **Extension** | **No. cycles** | **Final extension** | **Reference** |
| *Giardia duodenalis* | *ssu* rRNA | | 95°C 15 min | 95°C 15 s | 60°C 1 min | 72°C 30 s | 45 | – | 55 |
|  | *gdh* | | 95°C 3 min | 95°C 30 s | 55°C 30 s | 72°C 1 min | 35 | 72°C 7 min | 56 |
|  | *bg* | | 95°C 7 min | 95°C 30 s | 65/55°C 30 s | 72°C 1 min | 35 | 72°C 7 min | 57 |
|  | *tpi* | | 94°C 5 min | 94°C 45 s | 50°C 45 s | 72°C 1 min | 35 | 72°C 10 min | 58 |

*bg*: β-giardin; *gdh*: Glutamate dehydrogenase; *ssu* rRNA: Small subunit ribosomal RNA; *tpi*: Triose phosphate isomerase.
